# A proof of concept study on real-time LiMAx CYP1A2 liver function assessment of donor grafts during normothermic machine perfusion

**DOI:** 10.1038/s41598-021-02641-0

**Published:** 2021-12-06

**Authors:** Ivo J. Schurink, Jubi E. de Haan, Jorke Willemse, Matteo Mueller, Michael Doukas, Henk Roest, Femke H. C. de Goeij, Wojciech G. Polak, Jan N. M. Ijzermans, Philipp Dutkowski, Luc J. W. van der Laan, Jeroen de Jonge

**Affiliations:** 1 Division of Hepatopancreatobiliary Surgery, Department of Surgery, Erasmus MC Transplant Institute, Erasmus University Medical Center, Rotterdam, The Netherlands; 2 Department of Intensive Care Medicine, Erasmus University Medical Center, Rotterdam, The Netherlands; 3 Department of Surgery, University Hospital of Zürich, Zürich, Switzerland; 4 Department of Pathology, Erasmus University Medical Center, Rotterdam, The Netherlands

**Keywords:** Biomarkers, Gastroenterology, Medical research

## Abstract

No single reliable parameter exists to assess liver graft function of extended criteria donors during ex-vivo normothermic machine perfusion (NMP). The liver maximum capacity (LiMAx) test is a clinically validated cytochromal breath test, measuring liver function based on 13CO2 production. As an innovative concept, we aimed to integrate the LiMAx breath test with NMP to assess organ function. Eleven human livers were perfused using NMP. After one hour of stabilization, LiMAx testing was performed. Injury markers (ALT, AST, miR-122, FMN, and Suzuki-score) and lactate clearance were measured and related to LiMAx values. LiMAx values ranged between 111 and 1838 µg/kg/h, and performing consecutive LiMAx tests during longer NMP was feasible. No correlation was found between LiMAx value and miR-122 and FMN levels in the perfusate. However, a significant inverse correlation was found between LiMAx value and histological injury (Suzuki-score, R = − 0.874, P < 0.001), AST (R = − 0.812, P = 0.004) and ALT (R = − 0.687, P = 0.028). Furthermore, a significant correlation was found with lactate clearance (R = 0.683, P = 0.043). We demonstrate, as proof of principle, that liver function during NMP can be quantified using the LiMAx test, illustrating a positive correlation with traditional injury markers. This new breath-test application separates livers with adequate cytochromal liver function from inadequate ones and may support decision-making in the safe utilization of extended criteria donor grafts.

## Introduction

Organ shortage remains a limiting factor in liver transplantation, with high drop-out rates and mortality on the waiting lists as a consequence. Compromised donor organs are increasingly used to resolve this problem. However, these extended criteria donor (ECD) organs bear risk factors, such as high donor body mass index (BMI), older age, prolonged cold ischemia time, donation after circulatory death (DCD), and elevated liver injury parameters. These result in higher complication rates and reduced graft survival after transplantation. In particular, the risks of primary non-function (PNF), early allograft dysfunction, and non-anastomotic strictures are feared^1,2^.

The unpredictable functional capacity of ECD grafts increases the threshold to accept these ECD grafts. In DCD donors, only 35% of the offered grafts are accepted for transplantation; this is in contrast to a utilization rate as high as 82% in donation after brain death (DBD) donors^3^.

To increase the poor utilization rates of ECD grafts, perfusion and perseveration techniques have been explored. Normothermic machine perfusion (NMP) provides the graft with physiological temperature and oxygenation to prevent ischemia^4^. However, more objective measures for liver graft functionality are also required.

Assessing these grafts mainly on liver injury markers, AST and ALT (released from hepatocyte compartment) and alkaline phosphatase and gamma-glutamyltransferase (released from the biliary compartment), does not properly indicate the future functional capacity of the grafts^5^. From the donor procedures, it is known that livers donated after a period of resuscitation may have elevated injury markers, but they can still have an adequate liver function after transplantation^6^. This is in contrast to steatotic livers, which may not have elevated damage markers but can have compromised liver function after transplantation^7,8^. These contradictory findings highlight the importance of determining the functionality of ECD grafts before transplantation. Liver metabolism could be an objective measure of liver functionality. However, it can only be assessed under physiological conditions^9^, and thus, NMP opens up possibilities for assessing liver functionality^5,10,11^.

Currently, hepatic function during NMP has been assessed by measuring lactate clearance and/or bile production, but the use of these parameters is controversial since evidence reveals that they cannot reliably distinguish between well- and poor functioning grafts^5,10^. Therefore, more reliable liver function assays are required.

Measurement of cytochrome p450 (CYP) enzyme activity could be used to assess core hepatocyte function. These enzymes clear various compounds, such as steroids, fatty acids, and xenobiotics^12^. The breakdown of these compounds can be measured, and, subsequently, the rate at which breakdown occurs reflects the functionality of the liver.

The cytochromal activity of the CYP1A2 enzyme can be measured using the liver maximum function capacity (LiMAx) test. This test, which is clinically validated for determining (future remnant) liver functionality before and after major liver surgery, can measure the breakdown of ^13^C-methacetin into paracetamol and ^13^CO_2._ Subsequently, the CO_2_ with the heavier ^13^C isotope can be measured in the exhaled gas and be compared to ^12^CO_2._ A LiMAx value can be calculated from the ^12^CO_2_:^13^CO_2_ ratio^13^. These LiMAx values can predict the outcomes of liver surgeries^13–15^; studies indicate that a LiMAx score above 315 µg/kg/h indicates normal liver function, whereas a score below 140 µg/kg/h indicates poor liver function, with 40% mortality risk after major liver surgery^13^.

We hypothesize that the LiMAx test is equally suitable for assessing the liver function of ECD donor livers during machine perfusion *ex-vivo*. This study aims to adapt and optimize this test for a NMP circuit and to compare the LiMAx scores to traditional injury and function markers.

## Methods

### Human liver grafts

Human research livers, which all Eurotransplant centers declined for transplantation, were included in this study. Upon decline, the next of kin of the organ donors provided informed consent to use the liver for research purposes, documented in the Eurotransplant database. Regional organ procurement teams procured the livers. They used the standard procurement protocol of rapid *in-situ* cooling of the donor organs with UW preservation fluid, and this was followed by procurement and storage on melting ice for transport. The medical ethical committee of the Erasmus Medical Center Rotterdam (MEC 2012-090) approved this study.

As a reference for normal liver function, we included an explant liver of a Crigler-Najjar patient at the time of transplantation in our institution. This rare inherited disorder affects the metabolism of bilirubin by the hepatocyte, resulting in non-hemolytic jaundice, but otherwise preserves the normal CYP1A2 metabolic function and liver histology. We obtained Informed consent from this patient, and after hepatectomy the explant liver was rapidly flushed *ex-situ* with cold UW and stored on ice.

### Normothermic machine perfusion of human livers

Prior to NMP, the hepatic artery, the portal vein, and the bile duct were cannulated. NMP was performed with the Liver Assist device (Organ Assist, Groningen, the Netherlands). The following fluids were added to the circuit: 5 units of donor-matched fresh frozen plasma (Omniplasma, Sanquin, Amsterdam, The Netherlands), 3 units of ABO-matched red blood cells (Sanquin, Amsterdam, The Netherlands), 40 ml of 10% calcium gluconate (B. Braun Medical BV, Oss, The Netherlands), 25,000 units of non-fractionated heparin (Leo Pharma BV, Amsterdam, The Netherlands), and 25–50 ml of 8.4% sodium bicarbonate to adjust the pH. An oxygen flow of 0.5 L/min was provided to the arterial and portal membrane oxygenator. The perfusion pressure was monitored and set on the hepatic artery and the portal vein at 70 and 8–11 mmHg, respectively.

### LiMAx test

**Figure 1:**
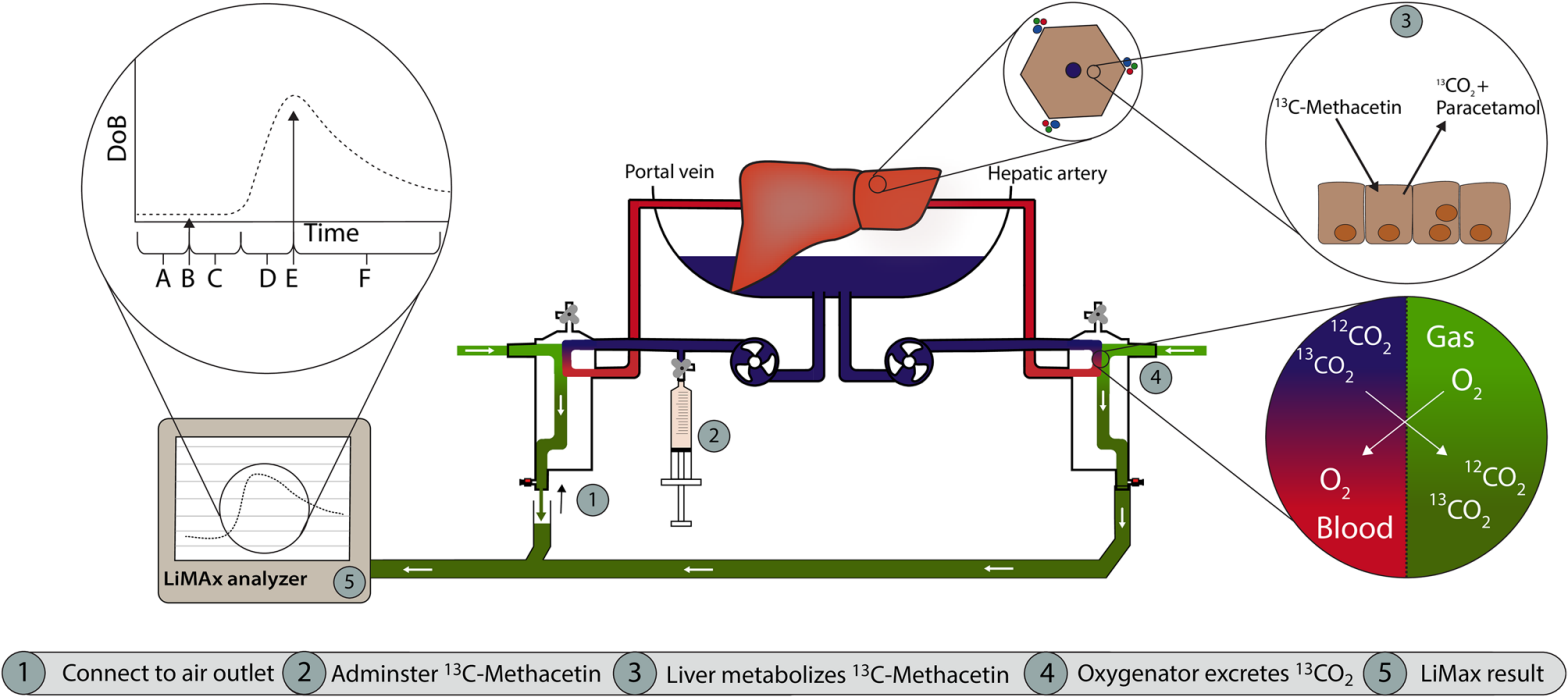
Overview of performing the LiMAx test in normothermic machine perfusion. (1) The LiMAx analyzer is connected to the air outlet of the membrane oxygenator. All extra air openings should be closed. (2) Then a baseline ^13^CO_2_:^12^CO_2_ ratio is measured, followed by administering the ^13^C-methacetin. (3) The ^13^C-methacetin is metabolized in the hepatocytes into ^13^CO_2_ and paracetamol. (4) The ^13^CO_2_ is excreted by the oxygenator from the perfusate into the “exhaled” air. (5) The LiMAx analyzer analyses the “exhaled” air, produces a curve illustrating delta over baseline (DoB) against the time in minutes. The LiMAx curve first presents the baseline that is being established (A), and then the ^13^C-methacetin is administered (B), followed by a delayed signal time (the time until the first increase in ^13^CO_2_ is measured by the analyzer; (C). Then, there is an acceleration phase (D), followed by the DoB_max_ (E), and finally resulting in an elimination phase (F) in which the signal slowly returns to the baseline.

The FLIP™ Analyzer (Humedics GmbH, Berlin, Germany) or the A2D Analyzer (ArgosMED GmbH, Karlsruhe, Germany) was connected to the gas outlets of the portal and arterial membrane oxygenators. All CO_2_ washed out by the membrane oxygenator was led through the air analyzer (see Fig. 1 for the schematic depiction).

The Crigler-Najjar explant liver was used as the gold standard for adjusting the LiMAx test for *ex-vivo* organ perfusion. The recommended dose of 2 mg ^13^C-methacetin (Humedics GmbH, Berlin, Germany)/ kg body weight was injected. Compared to standard testing, this resulted in a significantly prolonged time to reach the LiMAx value because of substrate overdosage. When 25% of the recommended dose was used, it yielded a LiMAx signal within the normal range of reaction time.

Instead of using the donor’s actual body weight to determine the dose of ^13^C-methacetin, we compensated for donor obesity by using the predicted lean metabolic donor body weight, according to Chan et al.^16^.

Integration of those two adjustments generated a new formula to calculate the dosage of ^13^C-methacetin during NMP:

1 $$^{{{13}}} {\text{C - methacetin}}\;{\text{dosage}} = LW^{\frac{4}{3}} *2.189*10^{ - 3}$$

The ^13^C-methacetin dosage is calculated in milligrams. The liver weight in grams is represented by LW. The used dosage of ^13^C-methacetin ranged between 34 and 71 mg.

The ^13^C-methacetin was administered 60 min after the start of NMP as a bolus injection to the portal circuit and followed by 20 ml of NaCl 0.9% flush. Before administration, a baseline ratio of ^13^CO_2_:^12^CO_2_ was recorded. The change in the ^13^CO_2_:^12^CO_2_ ratio was determined and displayed as delta over baseline (DoB). The duration of the LiMAx test was 60 min. Upon completion of the test, the LiMAx value was calculated according to the original formula^13^:

2 $$LiMAx= \frac{{DoB}_{max}*C*P*M}{\frac{1}{2}D}$$

The LiMAx values are calculated in µg/kg/h. In the formula, DoB_max_ represents the maximum of the DoB kinetics and C is a constant (C = 0.011237). Furthermore, P represents the estimated production rate of CO_2_ (300 mmol/h) per body surface in m^2^^17^, corrected for the liver’s estimated use of 17.52% of the resting energy expenditure^18^. Lastly, M is a constant that represents the molar mass of ^13^C-methacetin (M = 166.19 g/mol), and D represents the ^13^C-methacetin dosage in milligrams.

The method for determining the half-life of ^13^C-methacetin is indicated in the supplementary methods.

### Sequential LiMAx testing

Consecutive liver function measurements were performed to determine whether repetitive LiMAx tests impact the test characteristics in a closed-loop machine perfusion circuit.

One liver was put on NMP for 31 h. After 1 h of NMP, the LiMAx testing began. During testing, 4 boluses of ^13^C-methacetin were administered with 6 h, on average, between each bolus. After the final bolus, the washout period was 12 h instead of 6 h. The first LiMAx value of the initial bolus was calculated as previously described. For the remaining 3 boluses, the DoB_max_ was corrected for the residual ^13^C-methacetin of the previous bolus. The method for calculating the corrected DoB_max_ during consecutive measurements is listed in the supplementary methods.

### Traditional injury markers

Details on tissue and perfusate sampling, histology, and methods for determining microRNA (miRNA) and Flavin mononucleotide (FMN) are included in the supplementary methods.

### Statistical analysis

Statistical analysis was performed using the IBM SPSS statistical software package, version 25 (IBM Corp., Armonk, USA) or Prism (version 8.0, GraphpadSoftware). Continuous variables were summarized as median values, and ranges and categorical variables were expressed in percentages. The normality of the variable distribution was tested using the Kolmogorov-Smirnoff test. Correlations between different variables were calculated using the Pearson correlation coefficient (R). Tests were considered statistically significant at a 2-sided P-value of < 0.05. The ALT, AST, and miR’s values at 2 h of NMP were used for analysis. For FNM, the value measured at 30 min was used for analysis since this is commonly used in literature^19^. The illustration figure was created using Adobe Illustrator (CC 2018, Adobe Inc., Mountain View, USA). All methods were performed in according with relevant guidelines and regulations of the Erasmus Medical Center Rotterdam and the Netherlands.

## Results

### Donor and liver perfusion characteristics

Eleven human liver grafts (seven DCD, three DBD, and one explant graft) were used in this study. Livers were declined because of high age, high BMI, the DCD donor type, or a combination of these conditions (Table 1). The median asystolic donor warm ischemia time (WIT, in DCD) and cold ischemia time (CIT) of the donor livers was 15 (10**–**18) min and 14 (7–65) h, respectively.

**Table 1:** Donor and graft characteristics.

Human liver graft	Donor type	Donor age	Active smoker	Medication induce CYP1A2	Donor BMI (kg/m2)	ALT (u/l)	AST (u/l)	WIT (min)	CIT (min)	ET-DRI	Reason declined for Tx
H1	Explant	28	No	No	20.9	29	17	15	30	1.4	N/A
H2	DCD	62	No	No	37.0	130	104	18	873	2.5	Age & BMI
H3	DBD	66	No	No	38.6	103	91	-	860	1.8	BMI
H4	DCD	63	Yes	No	35.1	31	46	11	3886	4.0	Age
H5	DCD	65	No	No	25.1	62	113	17	764	2.5	Age
H6	DBD	63	No	Amiodarone	25.1	46	46	-	1199	2.0	Massive atherosclerosis
H7	DCD	68	No	No	36.0	89	87	15	1084	3.1	Age & BMI
H8	DCD	67	No	No	22.2	52	38	10	552	2.9	Age
H9	DCD	46	No	No	26.6	145	152	12	560	1.7	Size mismatch
H10	DCD	71	No	No	25.0	15	26	10	728	3.0	Age
H11	DBD	54	Yes	No	33.6	49	29	-	444	1.4	Steatosis

At the onset of NMP, the flow in the hepatic artery and portal vein was 132 (15–360) ml/min and 220 (130–750) ml/min, respectively. Flow typically increased during NMP, and at the end of perfusion, the hepatic arterial and portal venous flow was 190 ml/min (55–920) and 665 (300–1,440) ml/min. Oxygenation of the perfusate was stable during the perfusion with the PO_2_ ranging from 395 to 570 mmHg. The PCO_2_ in the central venous blood was 25.7 (19.1–38.0) mmHg and remained stable during perfusion, indicating that the perfused liver did not change CO_2_ production over time.

### Assessing graft injury and function markers

The traditional injury markers ALT and AST were mainly released in the first 30 min of perfusion, followed by a plateau phase after 120 min (Supplementary Fig. S1A,B). Liver H4, with a peak ALT and AST of 10,251 and 15,287, was deliberately exposed to a long CIT of 65 h to trigger PNF.

To assess mitochondrial damage, the new mitochondrial injury marker FNM was determined in the perfusate. As with AST and ALT, FNM values showed large scattering between grafts during NMP, with a plateau phase after 180 min in all livers except graft H7 (Supplementary Fig. S1D). After 30 min of perfusion, we found a significant correlation of FMN with ALT (R = 0.808, P = 0.005) and AST (R = 0.672, P = 0.033).

As illustrated in Supplementary Fig. S1E,F, microRNA markers specific for hepatocyte damage (miR-122) and cholangiocyte damage (miR-222) were detected in the perfusate. MiR-122 demonstrated a pattern similar to ALT and AST, with a rapid increase in some livers (H2, H4, H8, H10, and H11) and low levels in others (H3, H5, H6, H7, and H9). MiR-122 values were significantly correlated with FMN values (R = 0.730, P = 0.017).

The cholangiocyte-specific damage marker miR-222 demonstrated no increase in the majority of the livers. However, in livers H2, H9, and H10 an increase was observed after 120 min. The miR-122 and miR-222 ratio at 120 min was 30.7 (6.8–157.7).

### Liver function

The baseline values of lactate in the perfusate before beginning NMP ranged between 3.3 and 7.5 mmol/l. Lactate levels peaked in the first hour after perfusion, after which all livers except H4 started to clear lactate progressively until the end of perfusion (Supplementary Fig. S1C). Relative lactate clearance at 120 min, calculated as the percentage of the peak lactate, was 19% (0–40%). In line with the literature, neither peak lactate values nor lactate clearance was significantly correlated with the levels of injury markers ALT or AST after 120 min of perfusion. During the 240 min of NMP, the maximum bile production was 3 ml; the majority of donor livers did not produce any bile.

### Histology

Some of the liver grafts were declined due to either the amount of steatosis or donor BMI. In our liver cohort, the amount of steatosis ranged from 0–80% (Table 2). The median Suzuki histological injury score at the end of perfusion was 2.5 (range 0–5; Table 2).

**Table 2:** Histology scores and metabolic evaluation.

Human liver graft	Steatosis (%)	Suzuki score (range 0–12)	LiMAx value (µg/kg/h)	Half-life time of ^13^C-methacetin (min)
H1	ND	ND	1838	–*
H2	30	4	421	–*
H 3	15	3	777	–*
H4	40	5	111	–*
H5	0	1	971	16
H6	5	1	1310	14
H7	5	2	1203	46
H8	0	0	1552	19
H9	60	2	1063	–*
H10	0	3	1073	49
H11	80	4	667	51

*Out of the 11 NMP procedures, in 5 cases the half-life time was not possible to calculated due to a small amount of data points after the DoB_max_.

**Figure 2:**
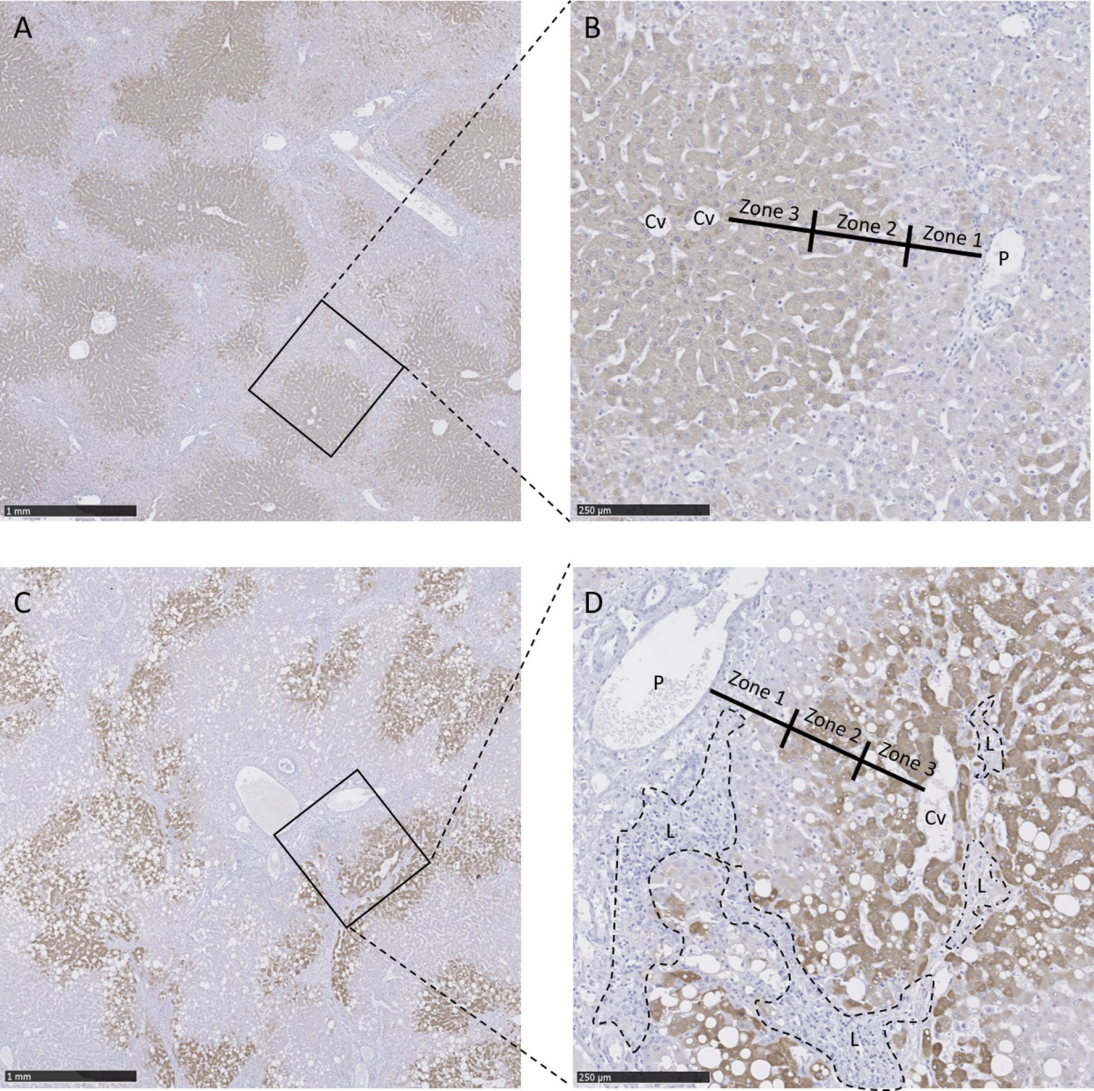
Histologic evaluation of biopsies from machine perfused livers stained with CYP1A2 immunohistochemistry. (**A**,**B**) Present liver H8 and panels C and D present liver H4. (**A**) and (**C**) consist of a low magnification picture (× 100). (**B**) and (**D**) display a high magnification (× 400). The portal vein (in the portal tract area) is represented by P, Cv represents the central vein, and L represents lymphocytic infiltrate. Zone 1, 2, and 3 represent the hepatic metabolic zones (as defined by the acinus of Rappaport). Zone 1 is the peri-portal zone, zone 2 is the intermediate zone, and zone 3 is the peri-central zone. H8 has normal liver microscopy; in this liver, it is visible that CYP1A2 is the cytoplasmatic hepatocellular stain highly present in zone 2 and 3, and it is less prominent in zone 1. H4 is microscopically a relatively injured graft. In this graft, lymphocytic infiltrates are present, causing hepatocyte loss and, therefore, loss of CYP1A2 positive cells.

CYP1A2 is only present in the hepatocytes (Fig. 2 & Supplementary Fig. S2). The distribution depends on the metabolic zonation; in metabolic zone 3 (peri-central) and zone 2 (intermediate), CYP1A2 is highly present. Compared to metabolic zones 3 and 2, CYP1A2 is expressed less in metabolic zone 1 (peri-portal). In H4, a lymphocytic inflammatory infiltrate is present. In these areas, the hepatocytes are replaced by lymphocytes, causing an overall reduction of CYP1A2 positive cells (Fig. 2B).

### Cytochromal liver function testing during NMP

**Figure 3:**
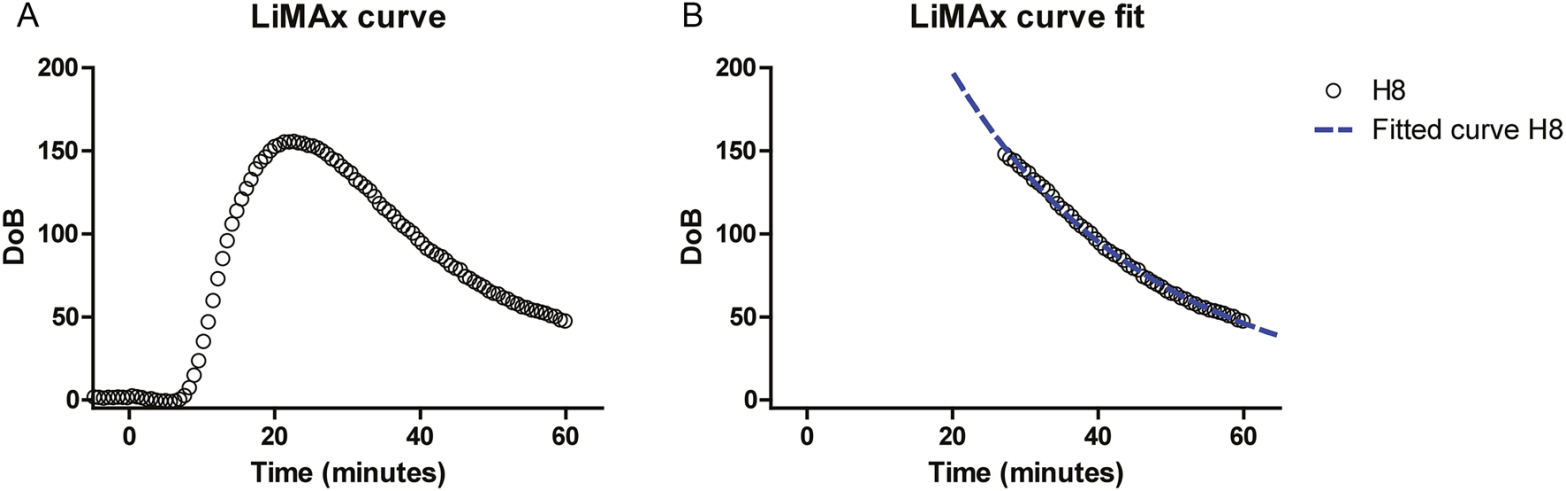
Feasibility of LiMAx testing during normothermic machine perfusion. Panel (**A**) displays the curve of the ^13^CO_2_:^12^CO_2_ ratio LiMAx curve as measured as a delta over baseline (DOB) during NMP. In the beginning, there is a flat line, which indicates the baseline, followed by the acceleration phase where the DoB increases until it reaches its maximum point (DoB_max)._ Then, there is a decline of the DoB in the elimination phase. Panel (**B**) presents the fit of formula 4 on the elimination phase of the curve, which is fitted with an R^2^ of 0.998. The K of the fitted formula was 3.633 × 10^–2^, which translates to a half-life time of 19 min.

After 60 min of NMP, the LiMAx test could be successfully performed in all livers. The amount of CO_2_ produced by the livers and the diffusion capacity of the oxygenator was sufficient enough to reach the detection level of the laser system. This resulted in a detectable ^13^CO_2_:^12^CO_2_ ratio with the subsequently calculated DoB LiMAx curve (Fig. 3A). After the start of the test, it took, on average, 7 min and 19 s (3:00–9:27) until the first signal was detected by the laser system. This signal delay time was caused by the initiation of the metabolic conversion in the liver, returning CO_2_ diffusion through the extracorporeal membrane oxygenation (ECMO), and filling the death space of the tubes to the analyzer. The signal delay was not correlated with the LiMAx value (R = 0.253, P = 0.453).

LiMAx values were in the range of 111–1838 µg/kg/h (Table 2). An adjusted ^13^C-methacetin dosage was tailored to ex-vivo use, as explained in the methods.

In total, the half-life times of ^13^C-methacetin could be calculated in six out of the 11 NMP procedures. The other five half-life times could not be calculated due to the small amount of data points after the DoB_max_, which can be solved by prolonging the breath analysis time. From the six half-life times, the R^2^ of the fitted formula to the raw data (Fig. 3B) ranged between 0.97 and 0.99. The half-life times ranged between 13 and 49 min (Table 2).

**Figure 4:**
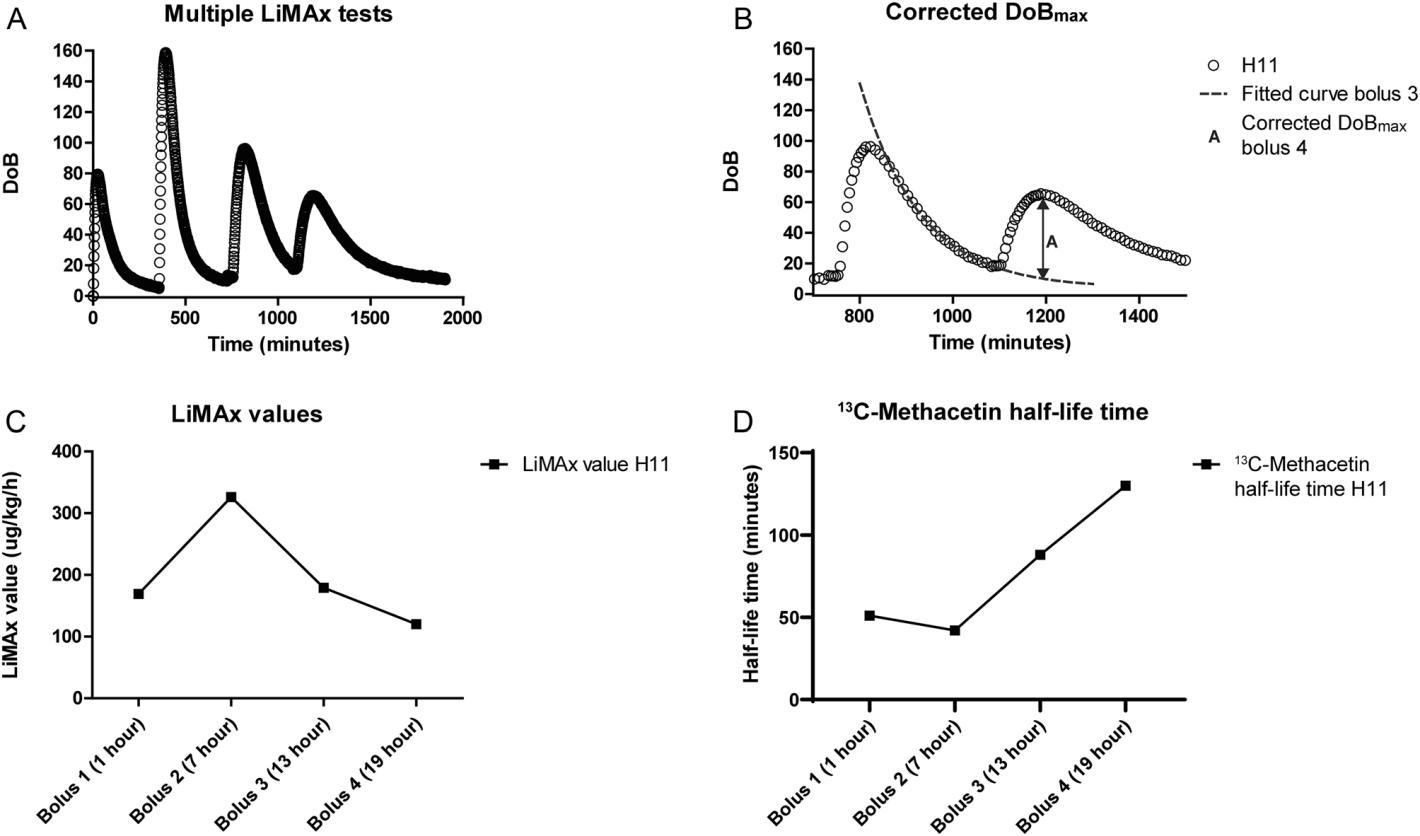
Consecutive LiMAx testing during normothermic machine perfusion. (**A**) Four sequentially repeated LiMAx tests over a period of 30 h of liver H11 were performed. Every 6 h, a new bolus methacetin was injected, resulting in a new DoB peak. (**B**) Before administering a new bolus, the DoB has not returned to the baseline, which should be corrected. The corrected DoB_max_ is made by calculating the remnant DoB of the previous bolus of ^13^C-methacetin and subtracting this from the new DoB_max._. (**C**) This illustrates the different LiMAx values in time during long-term machine perfusion. (**D**) This presents the different ^13^C-Methacetin half-life time during long-term machine perfusion.

In H11, long-term NMP with multiple LiMAx tests was performed (Fig. 4A,B). The first LiMAx value after 1 h of NMP was 677 µg/kg/h, which almost doubled after 6 h (1,304 µg/kg/h). Thereafter, LiMAx values decreased (718 and 480 µg/kg/h at 13 and 19 h respectively; Fig. 4C). The half-life time indicated an inverse pattern (Fig. 4D); after the first bolus at 1 h of NMP, the half-life time was 51 min, which decreased to 42 min at 7 h of perfusion. Thereafter, at 13 and 19 h of NMP, the half-life time increased to 88 and 130 min.

### Cytochromal liver function in relation to lactate clearance and injury markers

**Figure 5:**
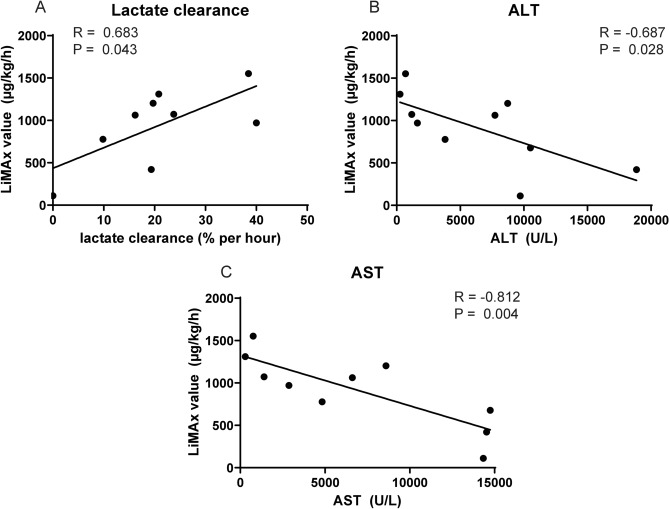
LiMAx value correlates to liver damage biomarkers. (**A**) Displays a significant correlation with lactate clearance. (**B**) Displays a significant correlation between LiMAx and ALT. (**C**) Expresses a significant correlation with AST.

A significant difference was found between lactate clearance (in percent/h) at 120 min and the LiMAx value (R = 0.683, P = 0.043; Fig. 5A). All livers except H4 cleared lactate. Liver H4, which was deliberately stored on ice for 65 h to guarantee primary non-function, had a LiMAx value of 111 µg/kg/h. The average LiMAx value of the livers that cleared lactate was 1,068 (421–1,838) µg/kg/h. Only two grafts had a lactate level lower than 2.5 mmol/l after 4 h. No significant differences were found in the LiMAx values between these livers and the liver grafts with a higher lactate level after 4 h (Supplementary Fig. S3A).

By correlating the LiMAx values as a measure of liver function to hepatocellular injury, we found a negative correlation with the markers ALT (R = − 0.687, P = 0.028) and AST (R = − 0.812, P = 0.004; Fig. 5B,C), indicating a relationship between injury and cytochromal liver function. No significant correlation was found between miR-122 (hepatocyte injury marker) and the LiMAx value (Supplementary Fig. S3B). However, a significant negative correlation with the specific cholangiocyte injury marker, miR-222 (Supplementary Fig. S3C), was identified. Furthermore, there was no clear correlation between FMN and the LiMAx value (Supplementary Fig. S3C).

**Figure 6:**
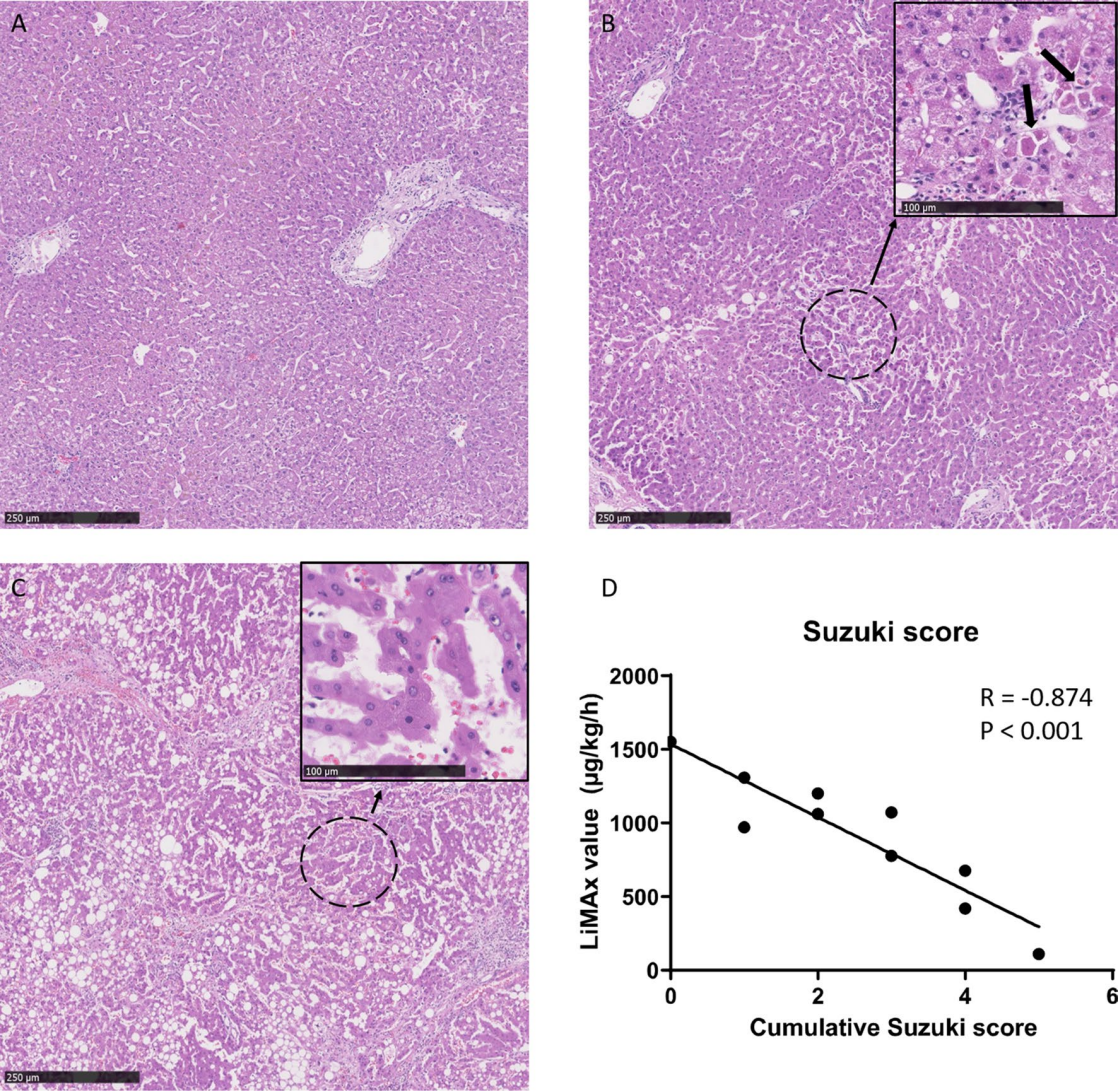
Histologic evaluation of biopsies from machine perfused livers with the Suzuki score. In (**A**–**C**), liver biopsies are stained with H and E staining from grafts with low, moderate, and high Suzuki scores. The pictures are magnified 400 times and the high magnification image is magnified 1,200 times. Panel A illustrates liver H8, which has a Suzuki score of 0, indicating normal liver microscopy with no congestion, vacuolization, or necrosis. (**B**) Illustrated H9, which has a Suzuki score of 2; some necrotic cells are indicated by the black arrows in the high magnification image. Furthermore, minimal vacuolization was identified. (**C**) Presents H4, which has a Suzuki score of 5. This liver was deliberately stored on ice for over 60 h. A moderate amount of congestion was found, as seen in the high magnification window. Furthermore, minimal vacuolization and some single-cell necrosis was noticed. (**D**) Presents a significant correlation of the Suzuki score with LiMAx score.

Regarding histological injury, three representative histological images are presented in Fig. 6A–C. At the end of NMP, a clear inverse correlation was observed between the LiMAx value and the Suzuki-score for histological injury (R = − 0.874, P < 0.001; Fig. 6D). No significant correlation was found between the LiMAx value and the degree of steatosis (Supplementary Fig. S3D).

## Discussion

Our results demonstrate the conceptual novelty of applying a breath-analysis test to the setting of *ex-vivo* NMP using the membrane oxygenator. In this study, we found marked differences in the outcomes between the functional LiMAx test and some hepatocyte and mitochondrial injury markers. This is supported by the clinical observation that livers demonstrating considerable injury during NMP may have positive post-transplant outcomes. Oppositely, livers with low or acceptable injury markers during NMP may experience poor initial function in the recipient^5^.

In current clinical practice, NMP is an emerging tool to evaluate donor livers before implantation^5^. The composition of produced bile during NMP has proven to be a good predictor of post-transplantation biliary complications^5,20^. However, to safely accept more ECD grafts, it is necessary to test the viability of both the biliary and the hepatocellular compartment, as the evaluation of the function of this compartment has proven to be more difficult.

Damage markers, such as ALT and AST, are typically used to assess the injury of the hepatocytes in the donor liver. Although liver damage and liver function are associated, they are not always correlated. Currently, to assess global liver function, lactate clearance is considered a function marker (e.g., reaching 2.5 mmol/l after 4 h of perfusion, as suggested from the Vittal trial)^11^. The problem with this value as a cut-off for adequate liver function is that starting (peak) lactate is a major determinant of reaching this level at 4 h. However, lactate level in NMP at 4 h is not only determined by the function of the donor liver but also the red blood packed cells; this is indicated by the lactate levels before the start of perfusion that ranges from 3.3 to 7.5 mmol/l. Furthermore, especially in a DCD donor, lactate accumulated inside the liver that is flushed into the NMP circuit may reflect a long agonal phase, without linear relation to injury or decreased liver function of this donor liver. Thus, absolute lactate levels at 4 h can underestimate the graft function in high starting lactate perfusions, while overestimating the graft function can result in PNF if the starting lactate is low^5^.

Therefore, our group advocates lactate clearance in the first 2 h of perfusion in percent/h as a better reflection of donor liver function during NMP, compared to absolute lactate levels at 4 h. This is underlined by our finding that there is a significant correlation between percent/h lactate clearance at 2 h and LiMAx value.

For optimal viability evaluation, a substrate test is preferable so that every liver is exposed to a similar amount of substrate, resulting in a more objective and comparable liver function result. This is underlined by our finding that severely steatotic (60–80%) livers can exhibit a decent LiMAx value and potentially be suitable for transplantation, while LiMAx values were lower in grafts without steatosis.

Currently, two substrate-based tests are used to evaluate liver function in patients: the indocyanine green (ICG) plasma clearance test and the LiMAx test^21^. The principle of the ICG test consists of the hepatic parenchyma eliminating ICG into bile in an unchanged form^22^. Since the elimination rate of ICG is highly dependent on blood flow and bile flow^23^, the ICG plasma clearance test does not exclusively reflect the hepatocellular compartment of the liver. The ICG plasma clearance is presumably a surrogate marker for the flow in the hepatic artery and the portal vein, which does not predict the post-transplantation outcome of a liver graft^5^. In contrast, the LiMAx test only reflects the capacity of the hepatocellular cytochromal metabolism. Therefore, we chose to study the LiMAx test in the NMP setting.

We based our hypothesis on the experience of Bednarsch et al., who demonstrated in patients supported by ECMO, that it is feasible to use LiMAx tests by connecting the membrane oxygenator to the LiMAx analyzer^24^.

The current range of Limax values in this study is broad, and this may limit translation to clinical practice. One explanation for this range is that the amount of ^13^C-methacetin administered to the system was based on donor weight. Separate from giving an initial overdose and tailoring this in the consecutive experiments, as explained in the methods, the relation between donor liver weight and donor weight was not linear, especially in this cohort with discarded livers. Steatosis affects both liver weight and liver function, independent from body (over)weight. In the future, this can be overcome by weighing every donor liver and administering ^13^C-methacetin according to the exact donor liver weight.

Another explanation for the broad range is that the low production of “normal” ^12^CO_2_ by the liver in an *ex-vivo* setting caused altered ^13^CO_2_ :^12^CO_2_ ratios when compared to patient settings where all organs and tissue contribute to the production of ^12^CO_2_. This can be corrected using equipment that can analyze the absolute (low) levels of CO_2_ produced by the liver and include this in the LiMAx formula. Such equipment will be available soon. However, NMP LiMAx values will still be in a different range from those identified in clinical practice with patients; this underlines the need for NMP specific cut-off values for poor and adequate liver function.

Alternatively, the half-life time of the ^13^C-methacetin, as a marker of CYP1A2 activity, might also be an adequate indicator of liver function during NMP, particularly since the half-life time of ^13^C-methacetin is independent of the ^13^CO_2_:^12^CO_2_ ratio and the dosage of ^13^C-methacetin. Recently, Holzhütter et al. demonstrated that the half-life of ^13^C-methacetin is a suitable discriminator and predictor for liver function in patients^25^. They reported a median half-life time of 16 min (6–48) in patients with normal liver function, whereas the half-life time in cirrhosis patients had extended to a median of 65 min (15–703)^25^. Half-life times in our cohort during NMP were in a similar range compared to clinical patients, suggesting that the half-life time can be used in the *ex-vivo* machine perfusion setting as well.

Different studies have demonstrated the reliability and accuracy of the LiMAx test to predict mortality and morbidity rate in a broad range of patients with acute and chronic liver diseases^13,15,26^. Therefore, we expected that the LiMAx test would be a suitable parameter to estimate global liver function in a NMP setting. In our study, we found a correlation between the LiMAx value and hepatocellular damage, histological score, and lactate clearance. While the literature associates graft steatosis with diminished liver function^27,28^, we did not find any correlation between the LiMAx values and the percentage of steatosis. This may have been caused not only by other factors, such as WIT, CIT, and donor type, that have a larger impact but also steatosis up to 60% may be compatible with adequate liver function, provided that other risk factors are minimal^29–31^. Our small heterogeneous cohort complicates drawing conclusions, and a larger follow-up study should investigate the relationship between LiMAx and steatosis in more detail.

The question remains as to what the optimal timing is to perform the LiMAx test. We chose for performance of the LiMAx test relatively early after reperfusion. The function of liver grafts on NMP improves within the first 2 h, as identified from clinical practice in parameters such as clotting times and bile production. Therefore, not all grafts that finally will show adequate function will meet the LiMAx test threshold at 1 h. However, if grafts do meet the criterion after 1 h, transplant logistics can be started, while the grafts that have not yet met the criterion could be re-examined at a later point during the NMP.

To confirm that the LiMAx test accurately reflects global liver function during NMP, the next step should be to transplant the assessed livers. This way, *ex-vivo* evaluation LiMAx values can be compared to relevant patient outcomes and a cut-off value for safe use can be established. Another interestingly future perspective of the presented technique is, that the principle of CO_2_ analysis of the graft is not restricted to measuring the function of the cytochromal P450 system. Other substrates might be useful to test other specific liver functions.

Finally, we would like to indicate the costs of the LiMAx test. While the initial purchase of a system is a considerable burden, costing around €50,000, the costs for individual tests are reasonable with €150 all-in. We feel that selective use in high-risk donor livers presents excellent cost-effectiveness if PNF is prevented and more livers can be accepted.

In conclusion, we demonstrated the proof of concept that liver function during *ex-vivo* normothermic machine perfusion can be objectively quantified using the LiMAx test. After determination of a cut-off level for safe use of ECD donor livers, the LiMAx test may be a valuable tool to aid decision-making for the acceptance of extended criteria donor liver grafts for liver transplantation.

## Supplementary Information

Supplementary Information.

## Abbreviations

ALT: Alanine aminotransferase
AST: Aspartate Aminotransferase
BMI: Body mass index
C: Carbon
CIT: Cold ischemia time
CYP1A2: 1A2 sub-enzyme of the cytochrome p450 family
DBD: Donation after brain death
DCD: Deceased after circulatory death
DoB: Delta over baseline
ECD: Extended criteria donor(s)
ECMO: Extracorporeal membrane oxygenation
FNM: Flavin mononucleotide
ICG: Indocyanine green
LiMAx: Liver maximum function capacity
NMP: Normothermic machine perfusion
miR: MicroRNA
PNF: Primary non-function
SCS: Static cold storage
WIT: Warm ischemia time
