# Supplementary material for: A proof of concept study on real-time LiMAx CYP1A2 liver function assessment of donor grafts during normothermic machine perfusion
Source: Sci Rep. 2021 Dec 6;11:23444. doi: 10.1038/s41598-021-02641-0 (PMC8648778; doi:10.1038/s41598-021-02641-0)
Supplement: Supplementary file 1 — Supplementary Information. [file 41598_2021_2641_MOESM1_ESM.pdf]

A proof of concept study on real-time LiMAx CYP1A2 liver function assessment of donor grafts during normothermic machine perfusion – supplementary method

Ivo J. Schurink, BSc, Jubi E. de Haan, MD, Jorke Willemse, MSc, Matteo Mueller, MD, Michael Doukas, MD, PhD, Henk Roest, PhD, Femke H.C. de Goeij, BSc, Wojciech G. Polak, MD, PhD, Jan N.M Ijzermans, MD, PhD, Philipp Dutkowski MD, PhD, Luc J.W van der Laan, PhD, Jeroen de Jonge, MD, PhD

Half-life time calculation of ^{13}C-methacetin

The metabolic conversion of CYP1A2 is a first order metabolic reaction (1). Therefore, the half-life time of ^{13}C-methacetin can be calculated by using DoB values. The half-life time is independent of the dosage of ^{13}C-methacetin administered.

In literature, the cumulative production of $^{13}CO_2$ equalizes the sum of all DoB over time (t) . This $\sum DoB(t)\ is$ formulated as follows (2):

(formula 3) $$\sum DoB(t) = \alpha - \alpha * e^{-K*t}$$

In this formula, α represents the limit of cumulative DoB, e represents Euler's constant (2.71828), K represents the first order rate constant and t represents the time in minutes.

After differentiating formula 3, formula 4 is created which describes the actual measured DoB after the DoB_{max}, reflecting the concentration of ^{13}C-methacetin still present:

(formula 4) $$DoB(t) = \alpha * K * e^{-K*t} + C$$

With use of Matlab (MathWorks, Natick, USA) the variable α and K of formula 4 were estimated by fitting on the raw DoB data. C represent a constant. The formula was fitted on the raw data points from the breath analyzer starting 5 minutes after the DoBmax until a minimum of 25 minutes after the DoBmax. The quality of the estimation of the variables was analyzed by calculating the coefficient of determination (R^2) of the formula on the data points.

With the estimated variable K from formula 4, the half-life time can be calculated as follows(2):

(formula 5) $$half\ life\ time = \frac{0.693}{K}$$

The half-life time is presented in minutes; 0.693 is the half-life constant. K represents the first order rate constant originating from formula 4.

Consecutive LiMAx testing at multiple time points correcting for the residual ^{13}C-methacetin

With the following formula the DoB_{max} is calculated during consecutive LiMAx testing, where is corrected for the surplus of the previous bolus methacetin:

(formula 6) $$Corrected\ DoB_{\max(N)} = DoB_{\max(N)} - DoB_{(N-1)}\left(t_{DoB_{\max(N)}}\right)$$

N represents the bolus number. $DoB_{max(N)}$ represents the maximum of the DOB kinetics after administration bolus N. $DoB_{(N-1)}\left(t_{DoB_{\max(N)}}\right)$ is DoB(t) with $t_{DoB_{\max(N)}}$ as variable of t. $t_{DoB_{max}(N)}$ represents the time in minutes until reaching the DoB_{max} of bolus N. The formula for calculating $DoB(t)$ is presented in formula 2; with this formula, the DoB value of the previous bolus (N-1) is calculated at the timepoint of the DoBmax of bolus N. Therefore, $DoB_{(N-1)}\left(t_{DoB_{\max(N)}}\right)$ represents the surplus DoB of the time point DoB_{max} of bolus N. With the corrected DoB_{max} the LiMAx value could be calculated using formula 3.

Sampling

During NMP, blood from the arterial oxygenator was collected every 30 minutes. Arterial and venous blood gasses were analyzed using the ABL90 FLEX PLUS blood gas analyzer (Radiometer Benelux BV, Zoetermeer, the Netherlands). Serum samples were stored at -80°C until analysis. Injury markers AST and ALT were measured in all samples in the clinical chemistry laboratory of the Erasmus MC according to standard protocol. At the start and at the end of NMP a wedge-biopsy was taken and fixed in 4% paraformaldehyde overnight to be further processed for paraffin-embedding according to standard procedures.

Histology

Paraffin embedded biopsies were sectioned at 5μm. Sections were stained with Hematoxylin-Eosin (HE) and liver injury was assessed using the Suzuki injury score(3). Furthermore, the sections were also immunohistochemistry stained for CYP1A2 with monoclonal CYP1A2 mouse anti-human antibody (OTI6E2; OriGene, Rockville, USA). The staining was performed accordingly the manufacture instructions.

RNA isolation, cDNA synthesis and Real-Time Quantitative PCR for miRNA

MicroRNA levels were determined following a previously published protocol(4). Total RNA was isolated from 200µL perfusate using the miRNEasy kit (Qiagen, Hilden, Germany), supplemented with 200 amol synthetic *Caenorhabditis elegans* miR-39 (Sigma Aldrich, Zwijndrecht, the Netherlands) during the lysis procedure. cDNA was synthesized using the Taqman microRNA Reverse Transcription Kit (ThermoFisher, Waltham, USA) in the presence of 6 IU Heparinase I (New England Biolabs, Ipswich, MA). Thereafter, miRNA detection levels were determined on an Applied Biosystems StepONE Plus Real-Time PCR machine and normalized using the levels of miR-39

FNM

Serum was diluted by 1/50 with NaCl 0,9% (B. Braun, Oss, The Netherlands), and 200µL of the diluted samples was analyzed with fluorescent measurement (Exitation at 485nm, Emission at 528nm) using a microplate reader (Cytation 3; Bio Tek Instruments GmbH, Lucerne, Switzerland).

References

1. Shiran MR, Lennard MS, Iqbal MZ, Lagundoye O, Seivewright N, Tucker GT, et al. Contribution of the activities of CYP3A, CYP2D6, CYP1A2 and other potential covariates to the disposition of methadone in patients undergoing methadone maintenance treatment. Br J Clin Pharmacol. 2009;67(1):29-37.
2. Singh S. Phatmacokinetics compartmental modeling. 1 ed. Shubham Vihar: Educreation; 2011.
3. Suzuki S, Toledo-Pereyra LH, Rodriguez FJ, Cejalvo D. Neutrophil infiltration as an important factor in liver ischemia and reperfusion injury. Modulating effects of FK506 and cyclosporine. Transplantation. 1993;55(6):1265-72.
4. Roest HP, Verhoeven CJ, de Haan JE, de Jonge J, JN IJ, van der Laan LJ. Improving Accuracy of Urinary miRNA Quantification in Heparinized Patients Using Heparinase I Digestion. Journal of Molecular Diagnostics. 2016;18(6):825-33.

A proof of concept study on real-time LiMAx CYP1A2 liver function assessment of donor grafts during normothermic machine perfusion – supplementary data

Ivo J. Schurink, BSc, Jubi E. de Haan, MD, Jorke Willemse, MSc, Matteo Mueller, MD, Michael Doukas, MD, PhD, Henk Roest, PhD, Femke H.C. de Goeij, BSc, Wojciech G. Polak, MD, PhD, Jan N.M Ijzermans, MD, PhD, Philipp Dutkowski MD, PhD, Luc J.W van der Laan, PhD, Jeroen de Jonge, MD, PhD

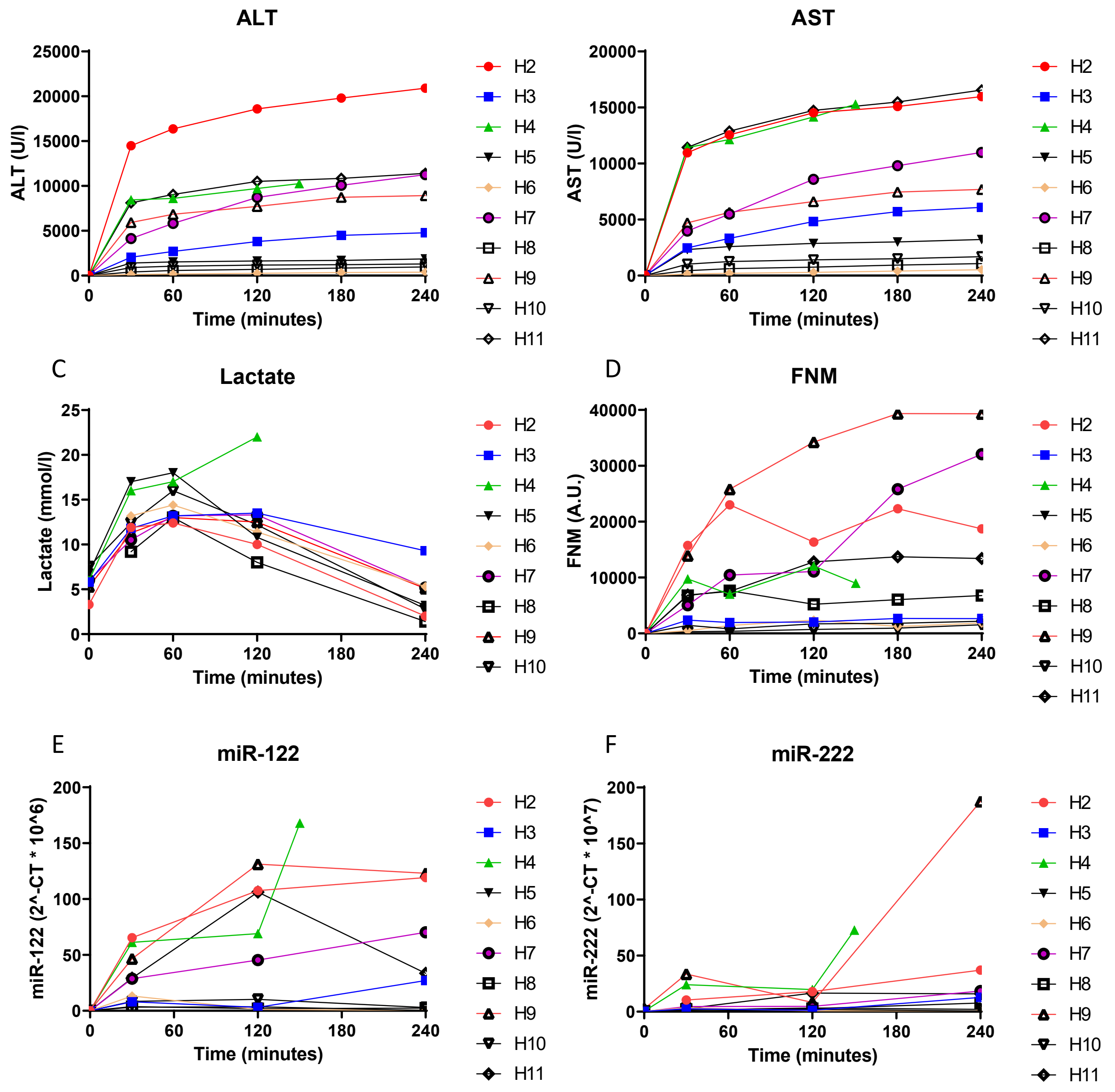

Supplementary figure 1. Kinetics of different biomarkers in perfusate during NMP. **A.** shows the ALT levels. **B.** shows the AST levels. **C.** shows the lactate levels. Lactate levels of H11 are not shown, due to measurement failure during the procedure. **D.** shows the FNM levels. **E.** shows the miR-122 levels. **F.** shows the miR-222 levels

Human liver 2:

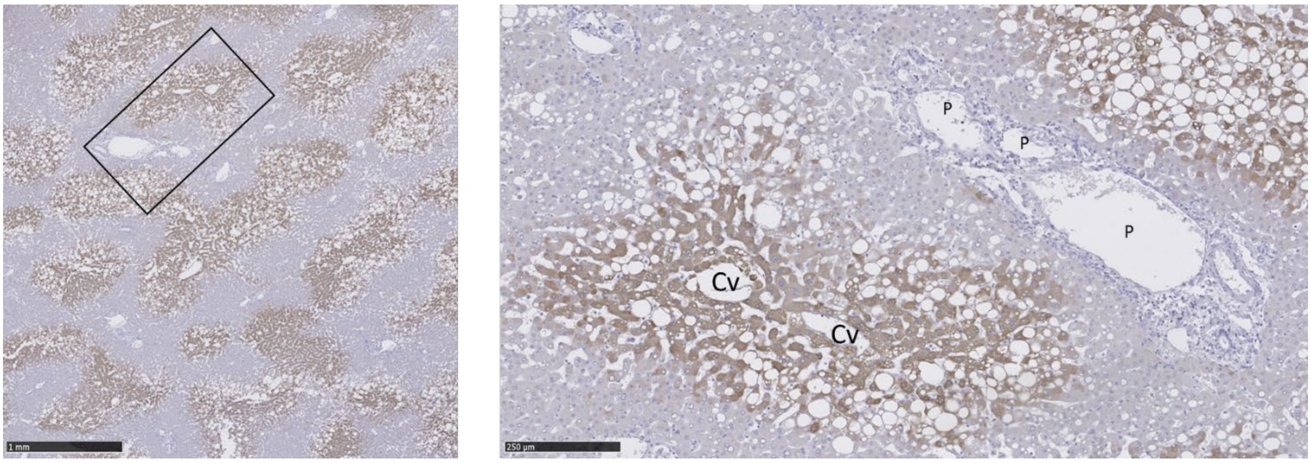

Human liver 3:

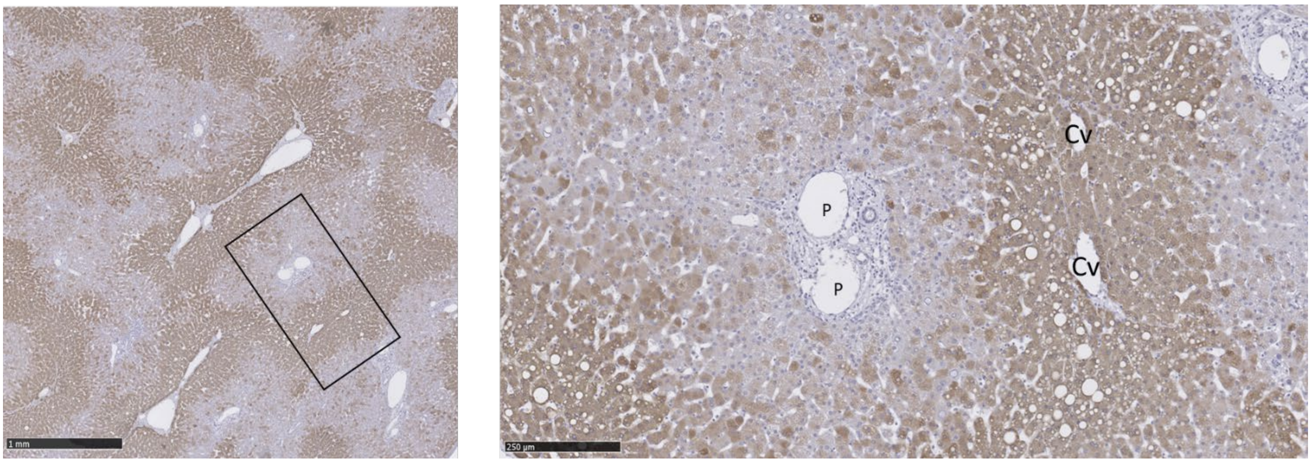

Human liver 4:

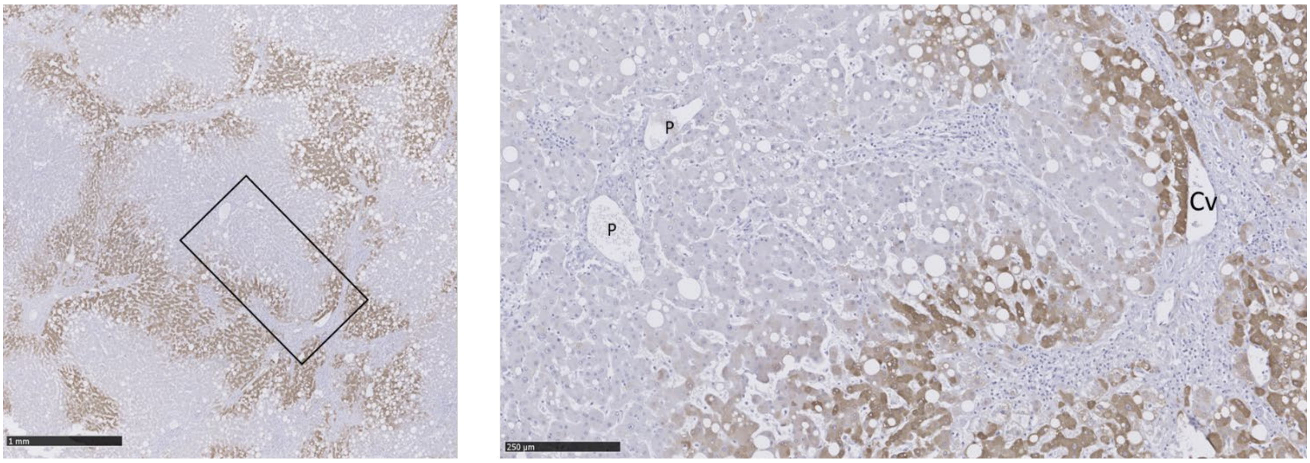

Human liver 5:

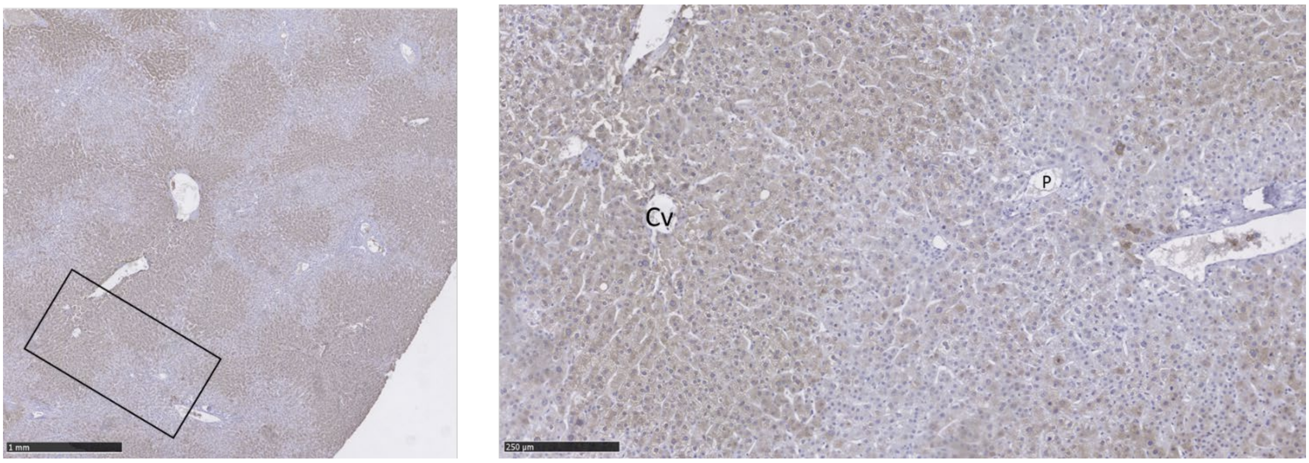

Human liver 6:

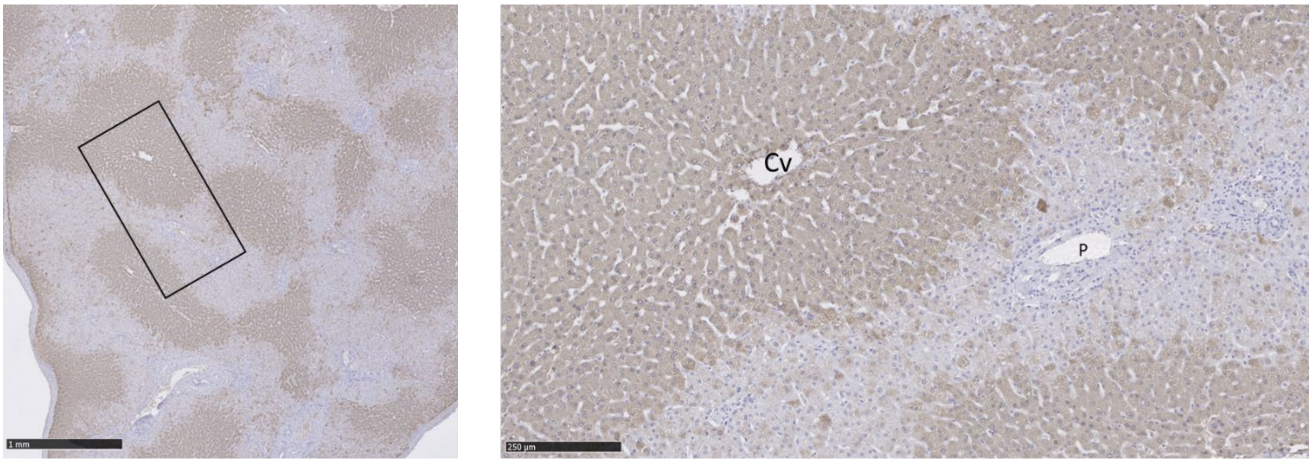

Human liver 7:

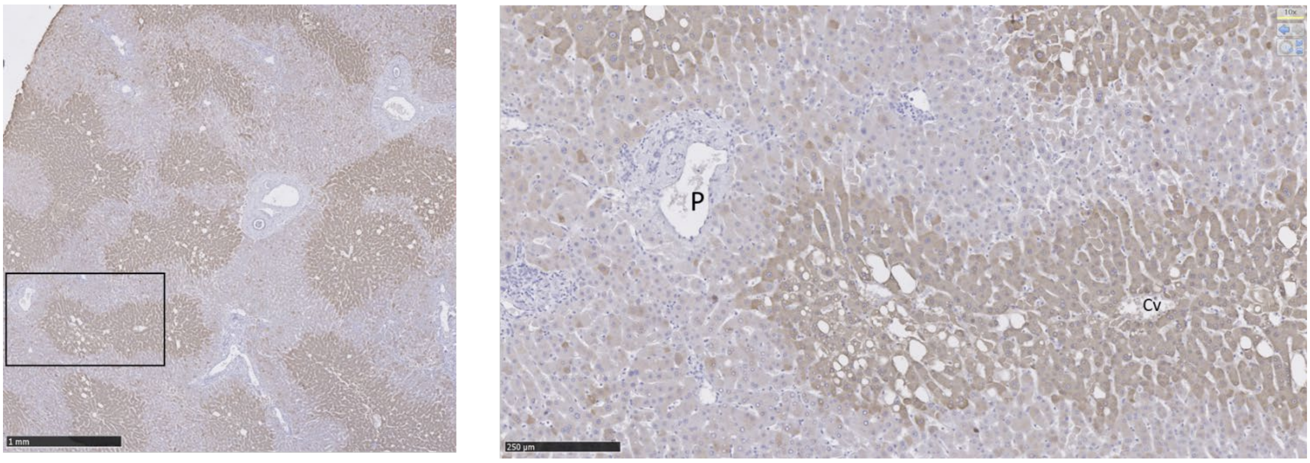

Human liver 8:

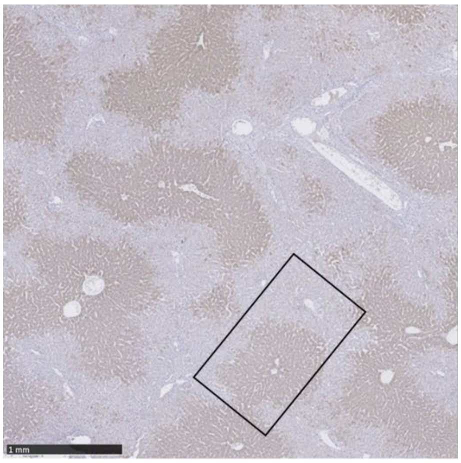

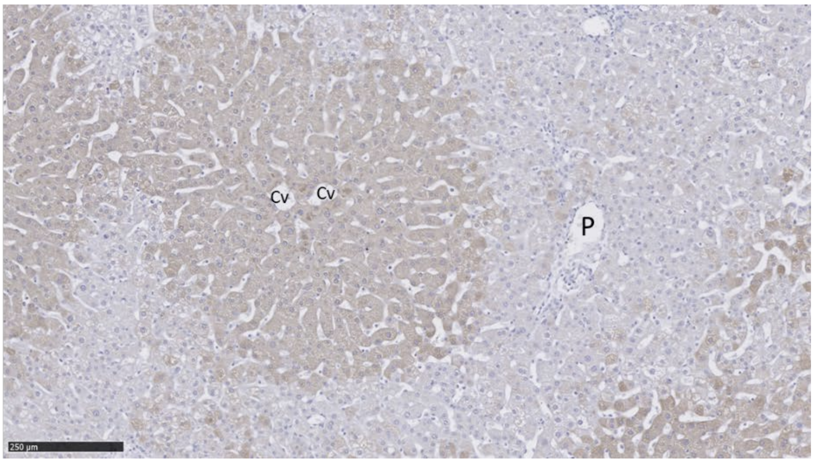

Human liver 9:

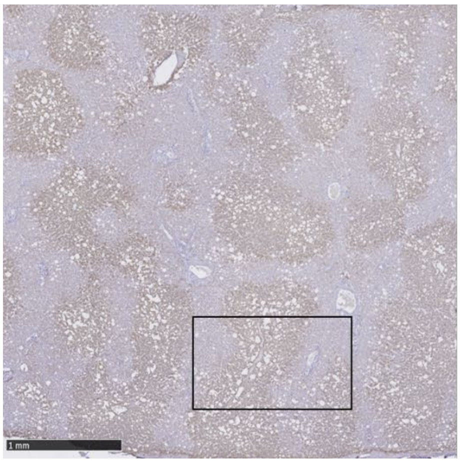

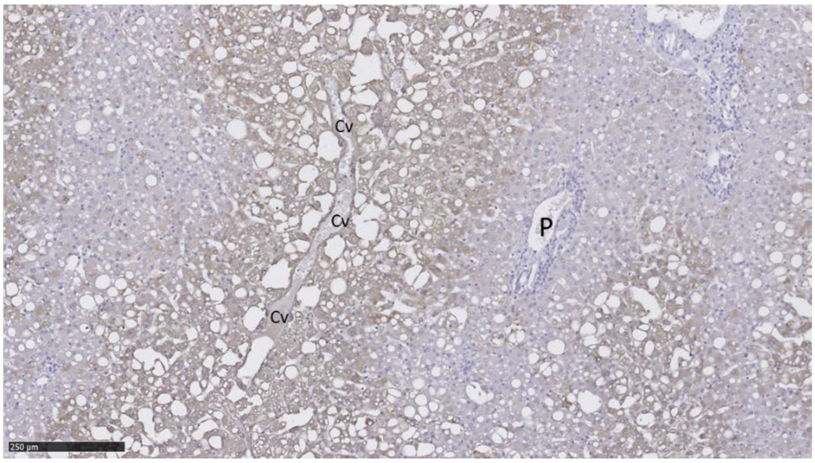

Human liver 10:

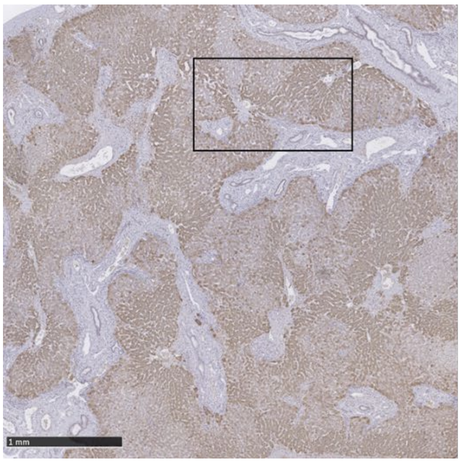

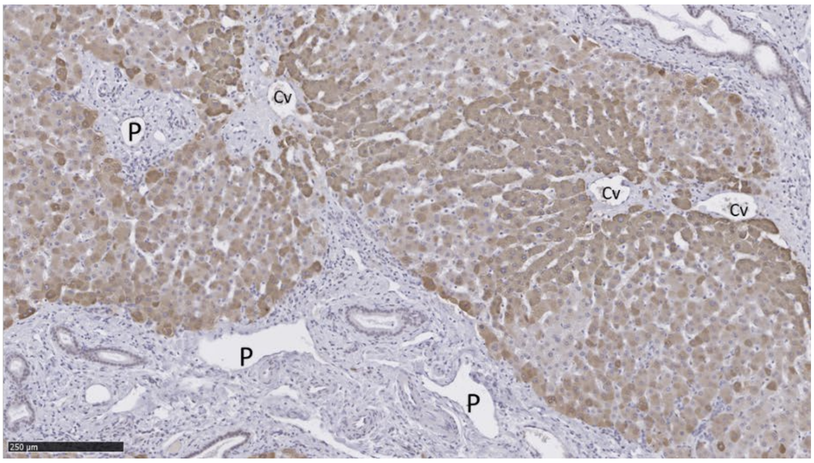

Human liver 11:

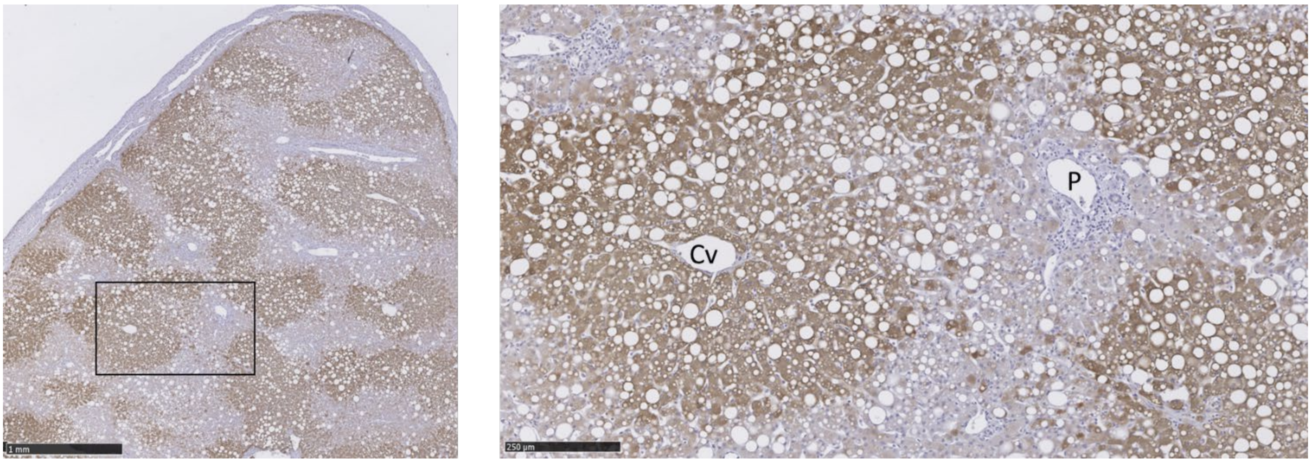

Supplementary figure 2: This figure shows the distribution of CYP1A2 in the liver. Livers H2 up to H11 are shown. The left pictures, which are the low magnification pictures, have a magnification of 100 time. The right pictures, which are the high magnification pictures, have a magnification of 400 times. All the high magnification picture show a portal vein (P) and a central vein (Cv). In between, the metabolic zonation of the liver is visible: zone 1 is the peri-portal zone, zone 2 is the intermediate zone, and zone 3 is the peri-central zone. CYP1A2 is mainly present in zone 2 and 3 and less present in zone 1. This distribution in zone 1 slightly differs between the different grafts.

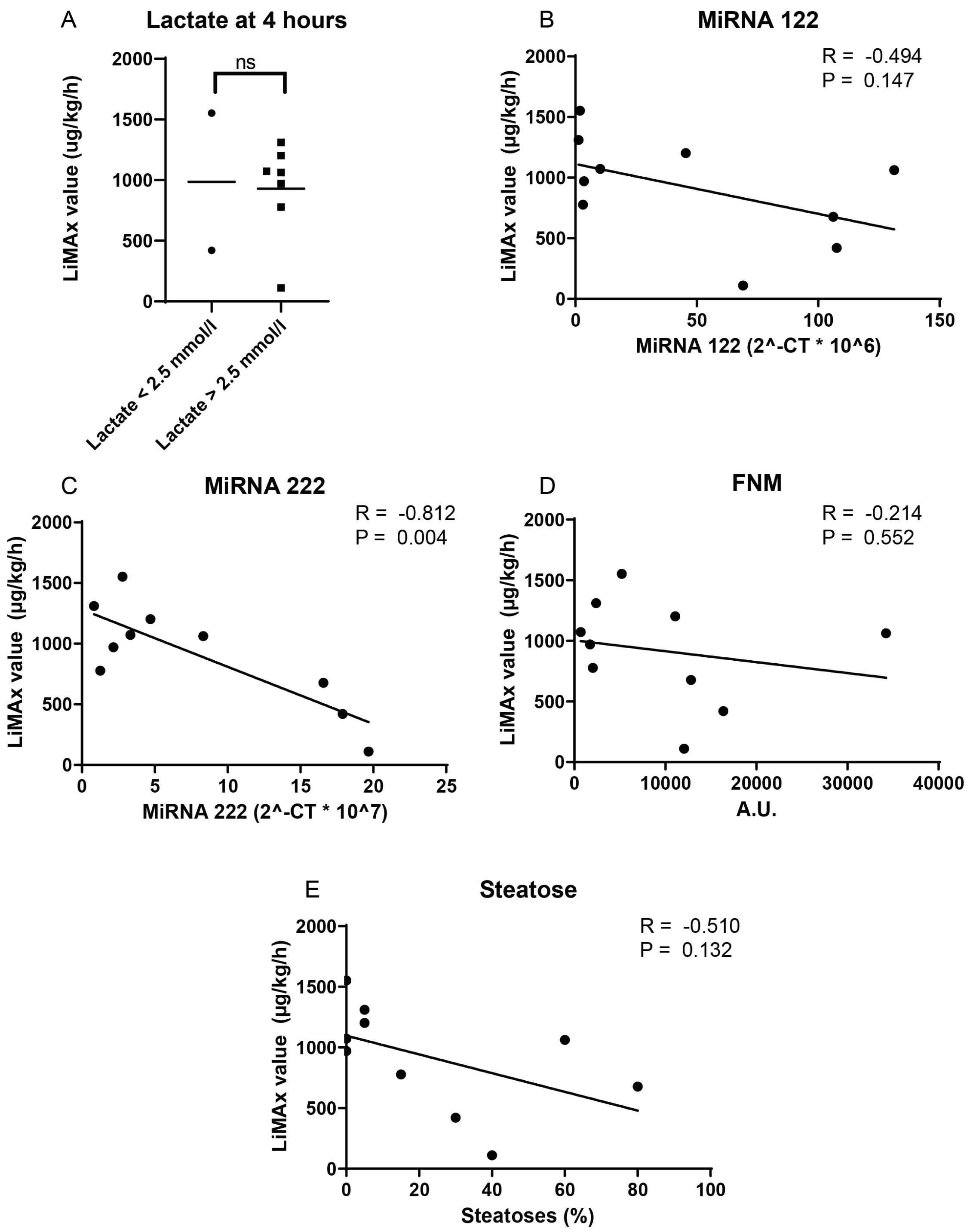

Supplementary figure 3: LiMAx value correlations to biomarkers **A**. LiMAx values of two groups, one group which had a lactate level of the perfusate under 2.5 mmol/l and the other group had a lactate level above 2.5 mmol/l at 4 hours of NMP. The LiMAx values did not differ significantly between these groups. **B.** shows that the LiMAx values and miRNA 122 are not correlated. **C** shows a significant correlation of the LiMAx values with cholangiocyte injury marker, miR-222 **D.** shows that the LiMAx values and FNM are not correlated. **E.** shows that the LiMAx values and the amount of steatosis are not correlated.
